# Unexpected Leiomyosarcoma 4 Years after Laparoscopic Removal of the Uterus Using Morcellation

**DOI:** 10.1155/2015/723606

**Published:** 2015-09-29

**Authors:** J. R. Prins, M. W. Van Oven, J. M. Helder-Woolderink

**Affiliations:** ^1^Department of Obstetrics and Gynaecology, Martini Hospital, P.O. Box 30033, 9700 RM Groningen, Netherlands; ^2^Department of Obstetrics and Gynaecology, University Medical Center Groningen, P.O. Box 30001, 9700 RB Groningen, Netherlands; ^3^Department of Pathology, Martini Hospital, P.O. Box 30033, 9700 RM Groningen, Netherlands

## Abstract

*Background*. Laparoscopic hysterectomies are increasingly popular; a morcellation device is often used. Although there are some clear benefits, morcellation of tissue does have potential risks. *Case Presentation*. In this case report we present a 55-year-old woman with an abdominal tumour 4 years after a laparoscopic hysterectomy using a morcellation device. Postoperative histological analysis, compromised by morcellated tissue, showed benign myoma. Because of the benign tumour no follow-up was performed. The patient presented now with an abdominal tumour, and she was scheduled for surgical removal of the tumour. During abdominal surgery the tumour appeared malignant and biopsies were taken. Histological analysis showed leiomyosarcoma, and the patient was referred to a third care centre for further treatment. The patient recovered quickly after abdominal removal of the tumour; however, after 7 months the patient had complaints and a CT scan showed a large intra-abdominal tumour with possible lung metastasis. The patient received palliative chemotherapy and died after 10 months. *Conclusion*. This case shows that although unexpected after a hysterectomy, a leiomyosarcoma has to be considered in case of a suspect tumour in the lower abdomen.

## 1. Introduction

Laparoscopic hysterectomies are increasingly popular because of the short recovery period of patients, causing short hospital stays and reducing healthcare costs [1–3]. In a laparoscopic-assisted supracervical hysterectomy (LASH) a morcellation device is often used. Besides “general” laparoscopic complications as bladder lesions, several studies have reported intra-abdominal pieces of the morcellated tissue remaining after the LASH [4–7]. Although remaining benign tissue will have relatively mild consequences, morcellation of malign tissue could have dramatic consequences leading to intra-abdominal metastasis. Therefore, uterine and cervical malignancies are considered as a contraindication for morcellation procedures. Furthermore, as morcellated tissue is very fragmented and reconstruction of an organ is not possible, histologic examination of morcellated tissue is challenging [8, 9]. Studies have shown that, in less than 0.5% of the patients having a LASH, an unexpected malignancy was found [10], with about 50% being a leiomyosarcoma (LMS). LMS is a rare uterine malignancy with a poor prognosis accounting for about 1-2% of uterine malignancies [11–13]. Usually women present themselves with abnormal vaginal bleeding, palpable pelvic mass, and pelvic pain [11, 12]. In this case report we present a 55-year-old woman with a LMS 4 years after a LASH using a morcellation device.

## 2. Case Presentation

A 55-year-old woman was referred to our clinic with malaise and an abdominal tumour. In 2009 she had a LASH procedure with use of a morcellation device for bleeding problems caused by a myomatous uterus. At the primary surgery the BMI of the patient was 27.7. As the patient was premenopausal the adnexa remained in situ. Postoperative histological analysis on the morcellated tissue (total 242 grams) showed benign myomas, with some mitotic activity and infarct type necrosis. Although the patient received adequate thrombotic prophylaxis by dalteparin, the patient developed postoperatively a deep-venous-thrombosis but recovered quickly after adequate treatment. As the tissue showed benign myomas no follow-up was performed. The patient was referred now with malaise, weight loss, and an abdominal mass. A vaginal ultrasound showed a large tumour (13 cm × 13 cm × 10 cm) most likely attached to the adnexa. There was no free fluid seen intra-abdominally. CA-125 and CEA were analysed to determine the malignancy risk (CA-125 14, CEA < 0.3). As the tumour markers were normal, the patient was scheduled for surgical removal of the tumour. During an abdominal surgery procedure 2 normal adnexa were seen, and the tumour appeared malignant and seemed connected to the remaining cervical tissue, the greater omentum, and sigmoid. No other abnormalities were observed intra-abdominally. As the tumour was connected to the omentum and sigmoid, and since the tumour bled easily, a malignancy was suspected and biopsies were taken for further diagnosis and the procedure was ended. A CT scan was performed and showed a large tumour without any evidence for intra-abdominal or thoracal metastasis. Histological analysis of the biopsies showed a smooth muscle tumour with high mitotic rates, tumour necrosis, and nuclear atypia, diagnostic for leiomyosarcoma. The patient was referred to a third care centre for surgical treatment and follow-up. She had abdominal removal of the tumour and showed a quick recovery. Because of the unexpected findings the original uterine tissue was reanalysed. Reanalysis of the morcellated uterine tissue showed features which confirmed those described in the original report. The patient received standard follow-up care, and after 7 months a CT scan was made because of abdominal complaints. The CT scan showed a large intra-abdominal tumour with possible lung metastasis. The patient received palliative chemotherapy and died after 10 months.

## 3. Discussion

A recent study showed that there is a small probability of unexpected malignancies in correctly prescreened patients for LASH procedures [10]. In this study in 0.25% of the patients having a LASH, an unexpected malignancy was found by histological analysis of the tissue obtained by the surgery [10]. About 50% of these unexpected malignancies were found to be a leiomyosarcoma (LMS) [10]. In this case report we present a woman with a LMS 4 years after an uncomplicated LASH procedure with the use of a morcellation device with no characteristics for malignancy in postoperative histological (re-)analysis.

Because of its clear advantages laparoscopic surgery becomes more and more popular and all sorts of technical devices are designed and used. Over years a morcellation device has been developed and has become a widely used tool, as it enables the possibility to reduce the size of tissue intra-abdominally [14, 15]. However, as morcellation causes intra-abdominal high speed rotation of tissue, several studies have reported intra-abdominal pieces of the morcellated tissue remaining after the morcellation procedure [4–7, 16, 17]. Although morcellation is regarded as a safe surgery tool, intra-abdominal dissemination of malignant cells could lead to higher mortality and morbidity. It has been shown that tumor morcellation during surgery increased the rate of abdominal-pelvic dissemination and adversely affected overall survival in patients with early uterine LMS during surgery [18]. Recently a case has been reported describing disseminated peritoneal leiomyosarcoma shortly after laparoscopic myomectomy with morcellation [19]. Contrary to our case, this woman showed multiple intraperitoneal mass lesions shortly after the morcellation procedure [19].

Recently, several statements have been released to discourage the wide spread use of morcellation and only offer this to appropriately screened, low risk women [20–22]. Although morcellation has clear advantages, the tissue obtained after morcellation of an organ is very fragmented and proper pathology examination is difficult [8]. The fragmented character of the morcellated tissue makes proper gross examination impossible and malignancy can be missed as a result of sampling error.

The effects of morcellation on pathology examination have been studied in a small study in which 10 women were included undergoing total hysterectomy without uterine morcellation; after pathology examination using standard techniques the uteri were deidentified, and all uteri were morcellated [9]. After morcellation the conclusion of the pathologist remained the same in 6 patients, whereas, in 4 the diagnosis was misclassified [9].

The fact that our patient got symptoms almost 5 years after her LASH, showing a single tumour without any (macroscopic) metastases, could suggest a malignant deterioration of nonmalign uterine cells spread during the morcellation procedure, or it could suggest a malignant deterioration of part of the cervical tissue which was not removed completely at the first surgery.

To summarize, the unexpected tumour origin in this case shows that tissue may remain after a macroscopic complete removal of an organ. The leiomyosarcoma in this patient could be a result of malignant deterioration of nonmalign uterine cells spread during the morcellation procedure, or malignant deterioration of remaining cervical tissue. Doctors should consider an unexpected cause in case of a suspect tumour in the lower abdomen.

## 4. Conclusion

This case report describes a woman with an unexpected leiomyosarcoma 4 years after the removal of the uterus without any macroscopic intra-abdominal metastasis. This case shows that a leiomyosarcoma always has to be considered in case of a suspect tumour in the lower abdomen, even after previous removal of the uterus.
